# Supplementary material for: Pancreatic beta-cell IL-22 receptor deficiency induces age-dependent dysregulation of insulin biosynthesis and systemic glucose homeostasis
Source: Nat Commun. 2024 May 29;15:4527. doi: 10.1038/s41467-024-48320-2 (PMC11137127; doi:10.1038/s41467-024-48320-2)
Supplement: Supplementary file 1 — Supplementary Information [file 41467_2024_48320_MOESM1_ESM.pdf]

Pancreatic Beta-Cell IL-22 Receptor Deficiency Induces Age-Dependent Dysregulation of Insulin Biosynthesis and Systemic Glucose Homeostasis

Haressh Sajiir[1, 2], Kuan Yau Wong[1, 2], Alexandra Müller[1, 2], Sahar Keshvari[1, 2], Lucy Burr[1, 2, 3], Elena Aiello[4], Teresa Mezza[5, 6], Andrea Giaccari [5, 7], Guido Sebastiani [4], Francesco Dotta[4, 8], Grant A. Ramm[2, 9], Graeme A. Macdonald[2, 10], Michael A. McGuckin[11], Johannes B. Prins[12], Sumaira Z. Hasnain*[1, 2, 13]

[1] Immunopathology Group, Mater Research Institute-The University of Queensland, Translational Research Institute, Brisbane, Australia

[2] Faculty of Medicine, The University of Queensland, Australia

[3] Department of Respiratory and Sleep Medicine, Mater Health, South Brisbane, Australia

[4] Diabetes Unit, Department of Medicine, Surgery and Neurosciences, University of Siena, Siena, Italy

[5] Dipartimento di Medicina e Chirurgia Traslazionale, Università Cattolica del Sacro Cuore, Roma, Italy

[6] Pancreas Unit, CEMAD Centro Malattie dell'Apparato Digerente, Medicina Interna e Gastroenterologia, Fondazione Policlinico Universitario Gemelli IRCCS, Roma, Italy

[7] Endocrinology and Diabetology Unit, Fondazione Policlinico Universitario Gemelli IRCCS, Roma, Italy

[8] Tuscany Centre for Precision Medicine (CReMeP), Siena, Italy

[9] QIMR Berghofer Medical Research Institute, Brisbane, Queensland, Australia

[10] Department of Gastroenterology and Hepatology, Princess Alexandra Hospital, Brisbane, Queensland, Australia

[11] School of Medicine, Dentistry and Health Sciences, University of Melbourne, Victoria, Australia

[12] Health Translation Queensland, Royal Brisbane and Women's Hospital, Herston, Australia

[13] Australian Infectious Disease Research Centre, University of Queensland, Brisbane, Australia

Correspondence to: Dr Sumaira Z. Hasnain, Address: Level 4/37 Kent St, Mater Research Institute-UQ, Translational Research Institute, Woolloongabba, Brisbane, QLD, Australia, Phone: +61 7 3443 6939, Email: sumaira.hasnain@mater.uq.edu.au

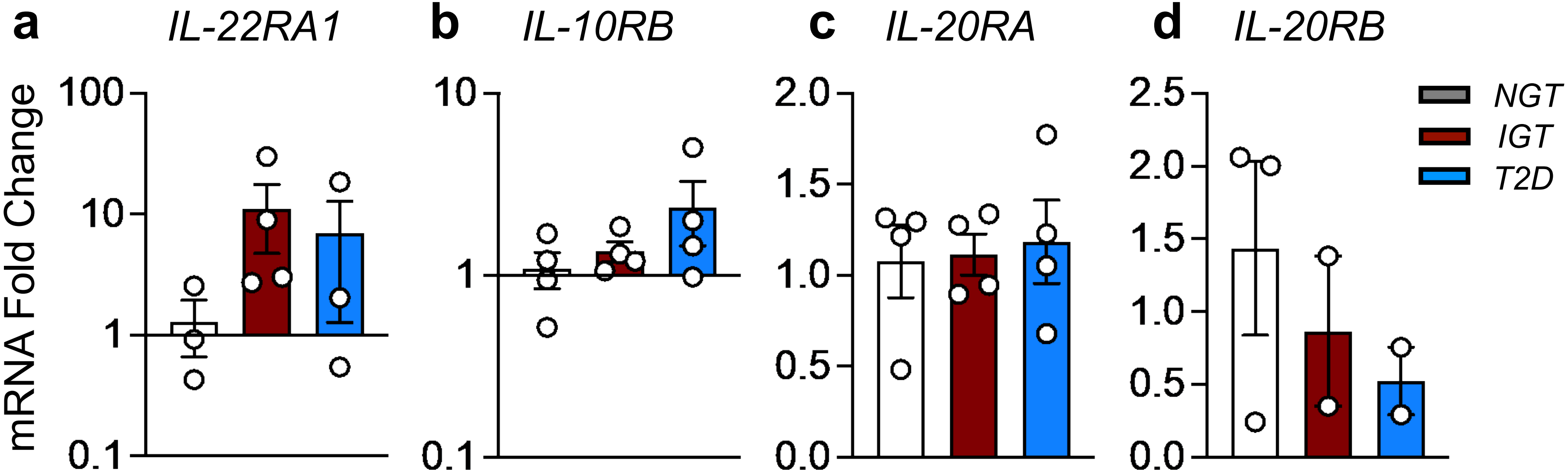

Supplementary Figure 1: In human donor islets, expression of IL-22 signaling genes increase with worsening glucose tolerance (a) mRNA fold change of *IL-22RA1*, **(b)** *IL-10RB*, **(c)** *IL-20RA*, and **(d)** IL-20RB gene expression in human islets relative to control (NGT) housekeeping gene *GAPDH*. All graphs are presented as Mean ± SEM. n = 4 biologically independent human islet donors ; Two-tailed Kruskal-Wallis test. Source data are provided as a Source Data file.

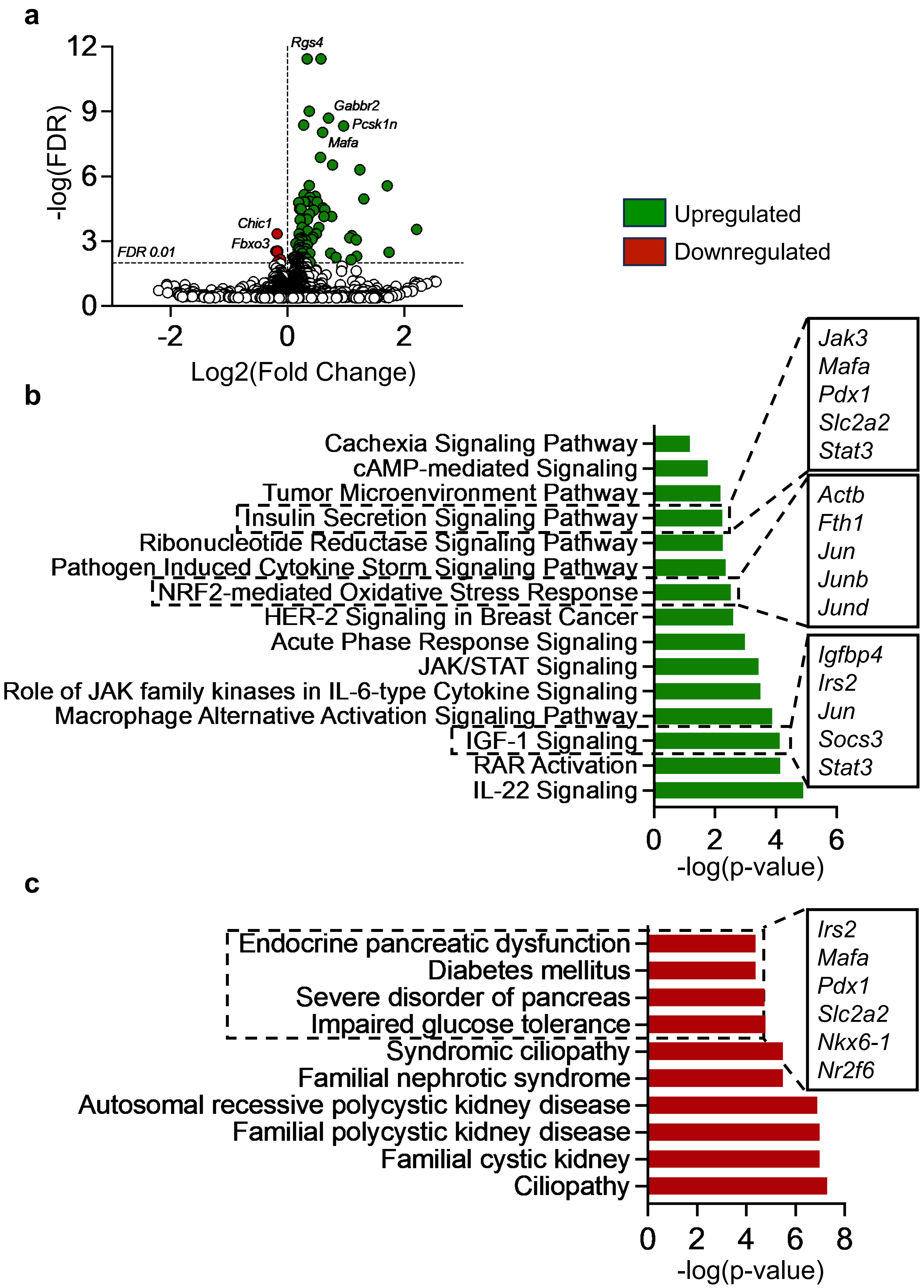

Supplementary Figure 2. Activation of IL-22ra1 in *MIN6N8* Pancreatic β-cells promotes insulin secretion signaling pathways. (a) Volcano plot for differentially regulated genes, **(b)** IPA pathway analysis for top 15 differentially regulated canonical pathways, highlighting the insulin secretion signalling pathway, IGF-1 signalling pathway, and NRF2-mediated Oxidative Stress Response, **(c)** Top 10 differentially regulated disease pathways generated via IPA machine learning software generated, FDR 0.01, in *MIN6N8* cells treated with PBS or IL-22 (50 ng mL^{-1}, 4h). n = 6 independent samples. Source data are provided as a Source Data file.

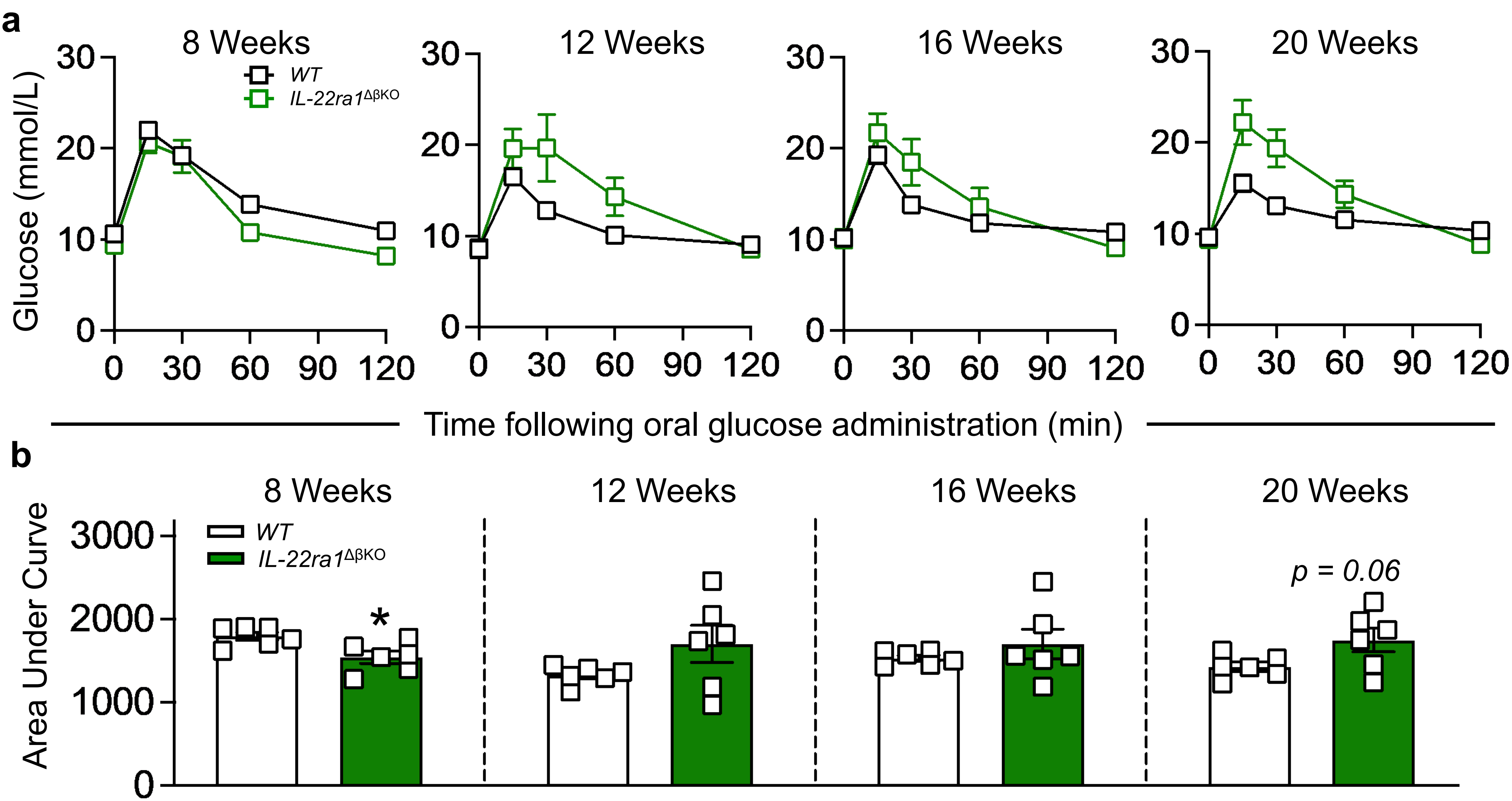

Supplementary Figure 3: In male animals, ablation of β-cell IL-22ra1 signaling leads to a less severe hyperglycemic phenotype with age (a) Changes in glucose tolerance following oral glucose administration in male animals with age. **(b)** Area under the curve during oral glucose tolerance tests in male animals with age (8 weeks, *p = 0.0162*). All graphs are presented as Mean ± SEM. Female animals; n = 6 biologically independent animals, Two-tailed unpaired Student's t-test. *p < 0.05; n.s., non-significant. *versus wildtype. Source data are provided as a Source Data file.

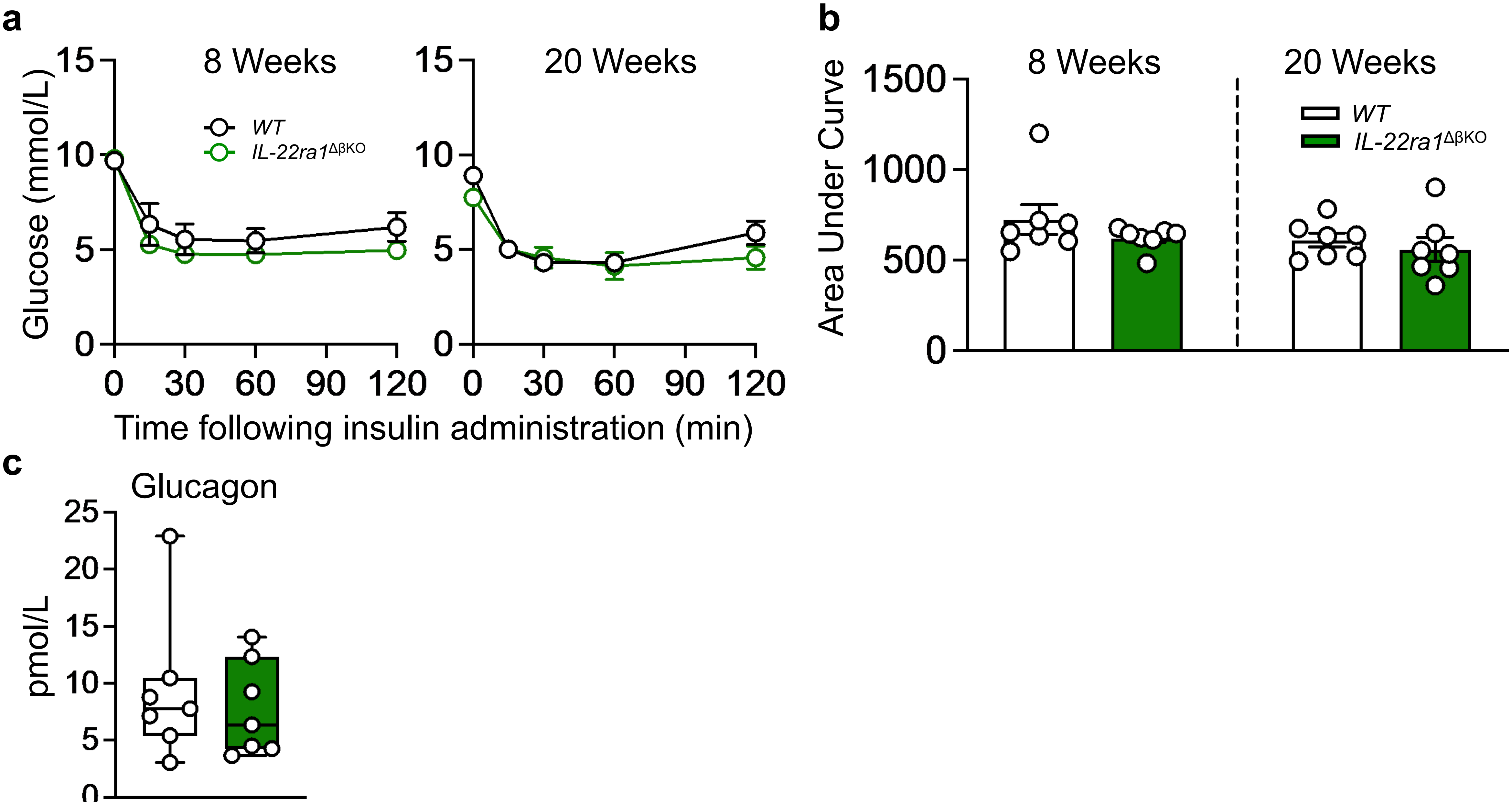

Supplementary Figure 4: Impaired glycemic tolerance due to ablation of pancreatic β-cell IL-22ra1 signaling is not caused by increased insulin resistance or impaired glucagon secretion (a) Changes in glucose levels following intraperitoneal insulin administration in animals with at 8 and 20 weeks of age. **(b)** Area under the curve during insulin tolerance tests in animals. **(c)** Serum glucagon levels at 20 weeks of age. All graphs in (**a-b**) are presented as Mean ± SEM, box plots in **(c)** display the median (central line), 25th to 75th percentile (box) and minimum to maximum values (whiskers). Female animals; n = 7 biologically independent animals, Two-tailed unpaired Student's t-test. n.s., non-significant. Source data are provided as a Source Data file.

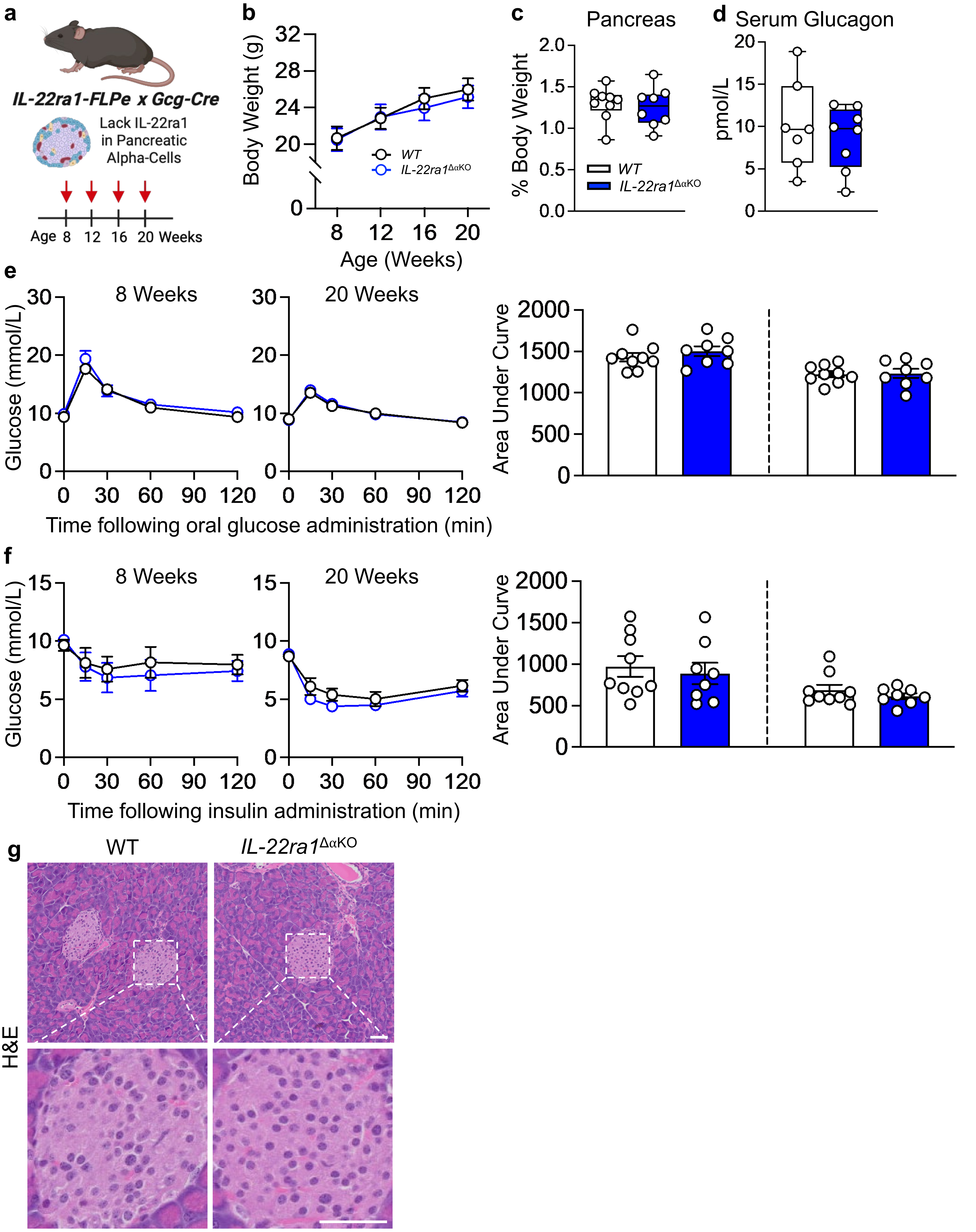

Supplementary Figure 5: Ablation of pancreatic α-cell IL-22ra1 signaling does not affect glycemic control or insulin sensitivity with age. (a) Experimental schematic created with BioRender.com, released under a Creative Commons Attribution-NonCommercial-NoDerivs 4.0 International license. **(b)** Changes in body weight with age and **(c)** pancreas weight as a percentage of total body weight in animals. **(d)** Serum glucagon at 20 weeks of age. **(e)** Changes in glucose levels and area under the curve following oral glucose administration in animals with age. **(f)** Changes in glucose levels and area under the curve following intraperitoneal insulin administration in animals with age. **(g)** H&E sections from pancreatic tissue. Box plots in **(c-d)** display the median (central line), 25th to 75th percentile (box) and minimum to maximum values (whiskers). All other graphs are presented as Mean ± SEM. Mixed genders; n = 8 biologically independent wildtype (*IL-22ra$^{fl/fl}$*) and 9 biologically independent *IL-22ra1$^{\Delta\alpha KO}$* animals. Two-tailed unpaired Student's t-test. n.s., non-significant. Source data are provided as a Source Data file.

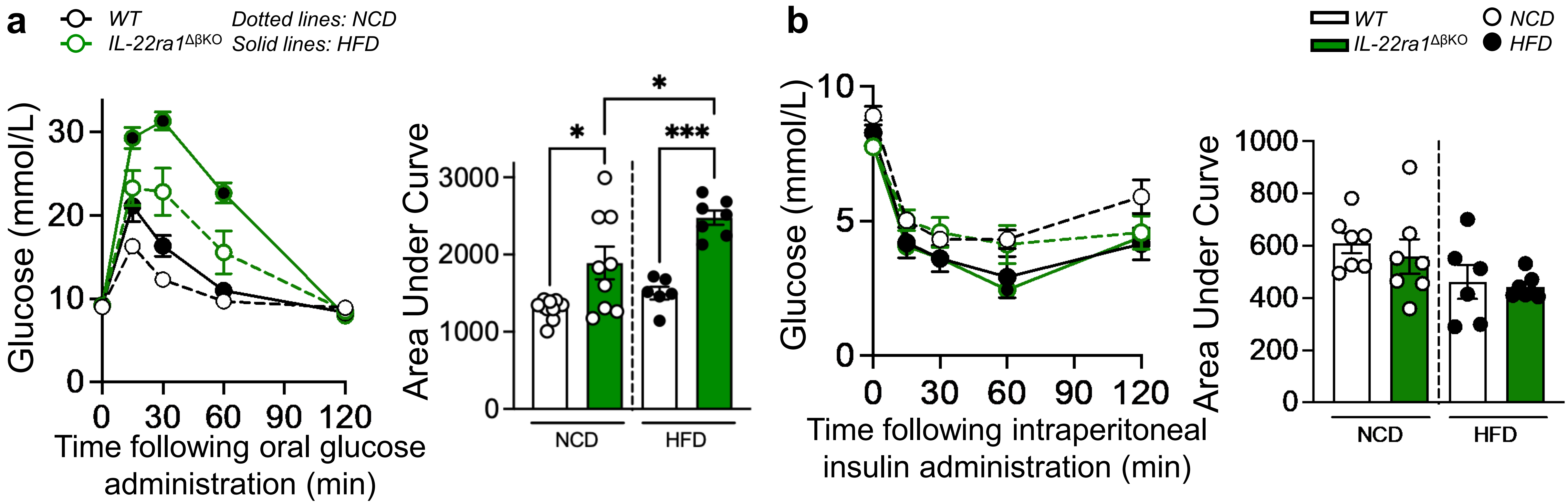

Supplementary Figure 6: Metabolic stress from high-fat diet feeding exacerbates impaired glycemic control in the absence of pancreatic β-cell IL-22ra1 signaling. (a) Changes in glucose levels and area under the curve following oral glucose administration, and **(b)** changes in glucose levels and area under curve following intraperitoneal insulin administration after 12 weeks of high-fat diet feeding. All graphs are presented as Mean ± SEM. Female animals; n = 7 biologically independent animals on NCD (wildtype *IL-22ra$^{fl/fl}$* and *IL-22ra1$^{\Delta\beta KO}$* mice), n = 6 wildtype (*IL-22ra$^{fl/fl}$*) and 7 biologically independent *IL-22ra1$^{\Delta\beta KO}$* animals on HFD. Two-tailed unpaired Student's t-test. *p < 0.05, **p < 0.01, ***p < 0.001; n.s., non-significant. Source data are provided as a Source Data file.

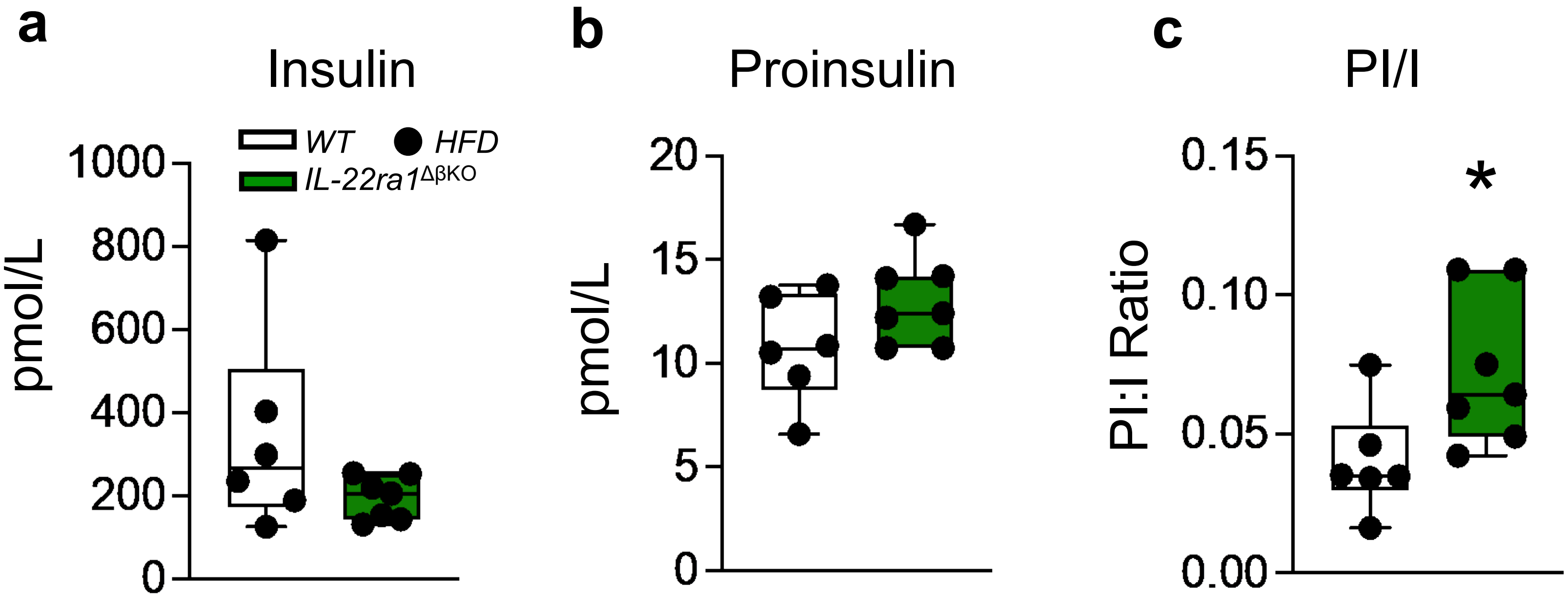

Supplementary Figure 7: Ablation of pancreatic β-cell IL-22ra1 signaling leads to impaired insulin quality following a HFD. (a) Total serum insulin, **(b)** proinsulin, and **(c)** proinsulin : insulin ratio in animals following 12 weeks of high-fat diet, *p = 0.0333*. Box plots display the median (central line), 25th to 75th percentile (box) and minimum to maximum values (whiskers). Female animals; n = 6 wildtype (*IL-22ra$^{fl/fl}$*) and 7 biologically independent *IL-22ra1$^{\Delta\beta KO}$* animals, Two-tailed unpaired Student's t-test. *p < 0.05, **p < 0.01, ****p < 0.0001; n.s., non-significant. *versus wildtype (*IL-22ra1$^{fl/fl}$*) HFD. Source Data are provided as a Source Data file.

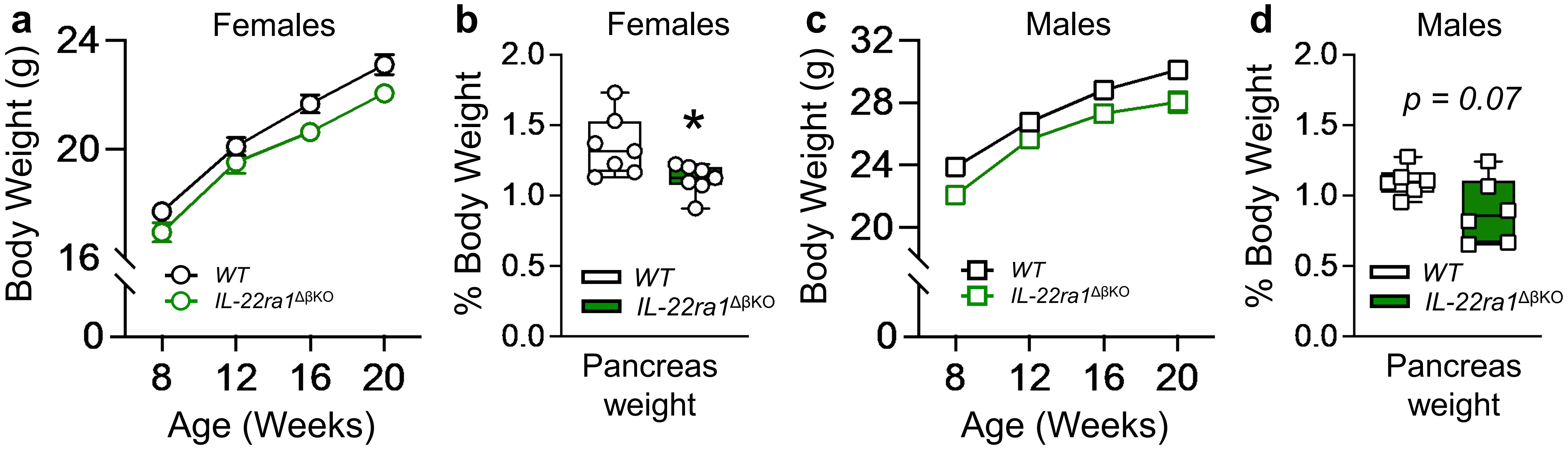

Supplementary Figure 8: Animals lacking β-cell IL-22ra1 signaling have smaller pancreata without significant weight loss (a) Changes in body weight with age and **(b)** pancreas weight as a percentage of total body weight in female animals, $p = 0.0223$. **(c)** Changes in body weight with age and **(b)** pancreas weight as a percentage of total body weight in male animals. Box plots in **(b, d)** display the median (central line), 25th to 75th percentile (box) and minimum to maximum values (whiskers), all other graphs are presented as Mean ± SEM. **(a-b)** n = 7 biologically independent female animals and **(c-d)** n = 6 biologically independent male animals. Two-tailed unpaired Student's t-test. *p < 0.05; n.s., non-significant. *versus wildtype (*IL-22ra1$^{fl/fl}$*) littermates. Source data are provided as a Source Data file.

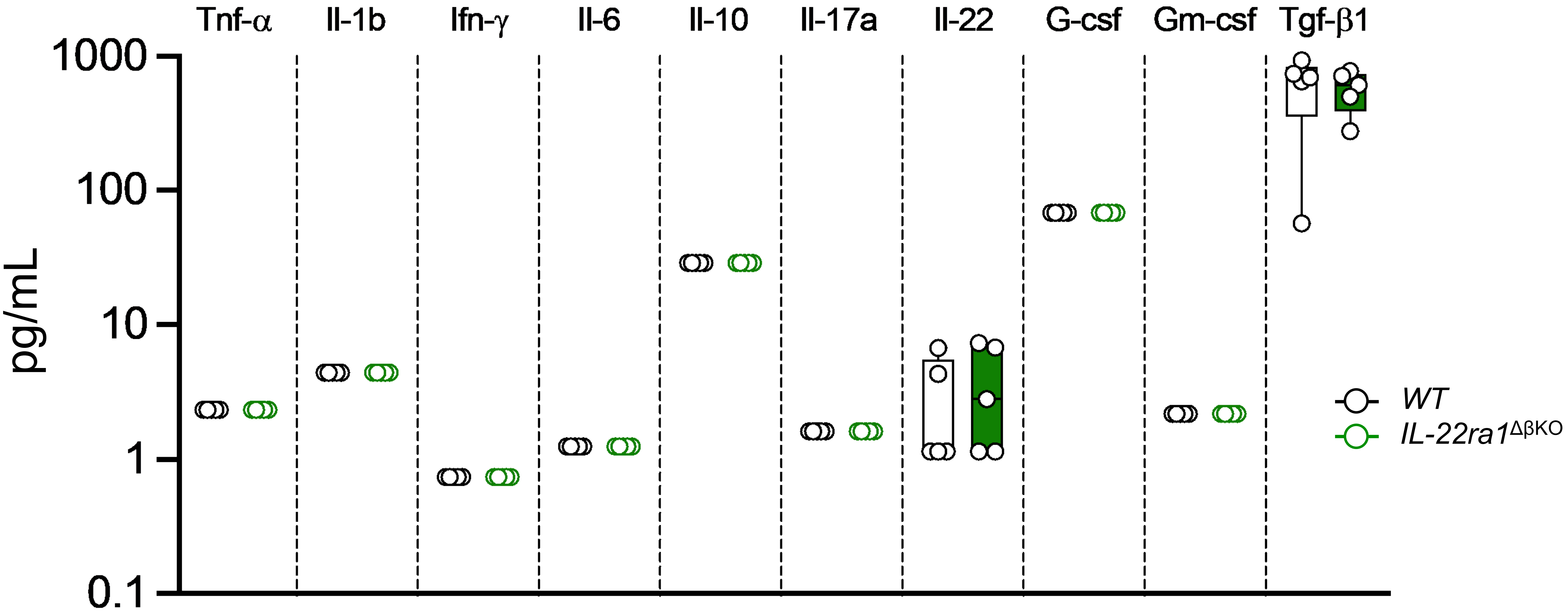

Supplementary Figure 9: Ablation of pancreatic β-cell IL-22ra1 signaling does not cause systemic inflammation. Changes serum Tnfa, Il-1b, Ifn-g, Il-6, Il-10, Il-17a, Il-22, G-csf, Gm-csf, and Tgf-b1 in mice at 20 weeks of age. Box plots in display the median (central line), 25th to 75th percentile (box) and minimum to maximum values (whiskers). Female animals; n = 5 biologically independent animals. Two-tailed unpaired Student's t-test. n.s., non-significant. Source data are provided as a Source Data file.

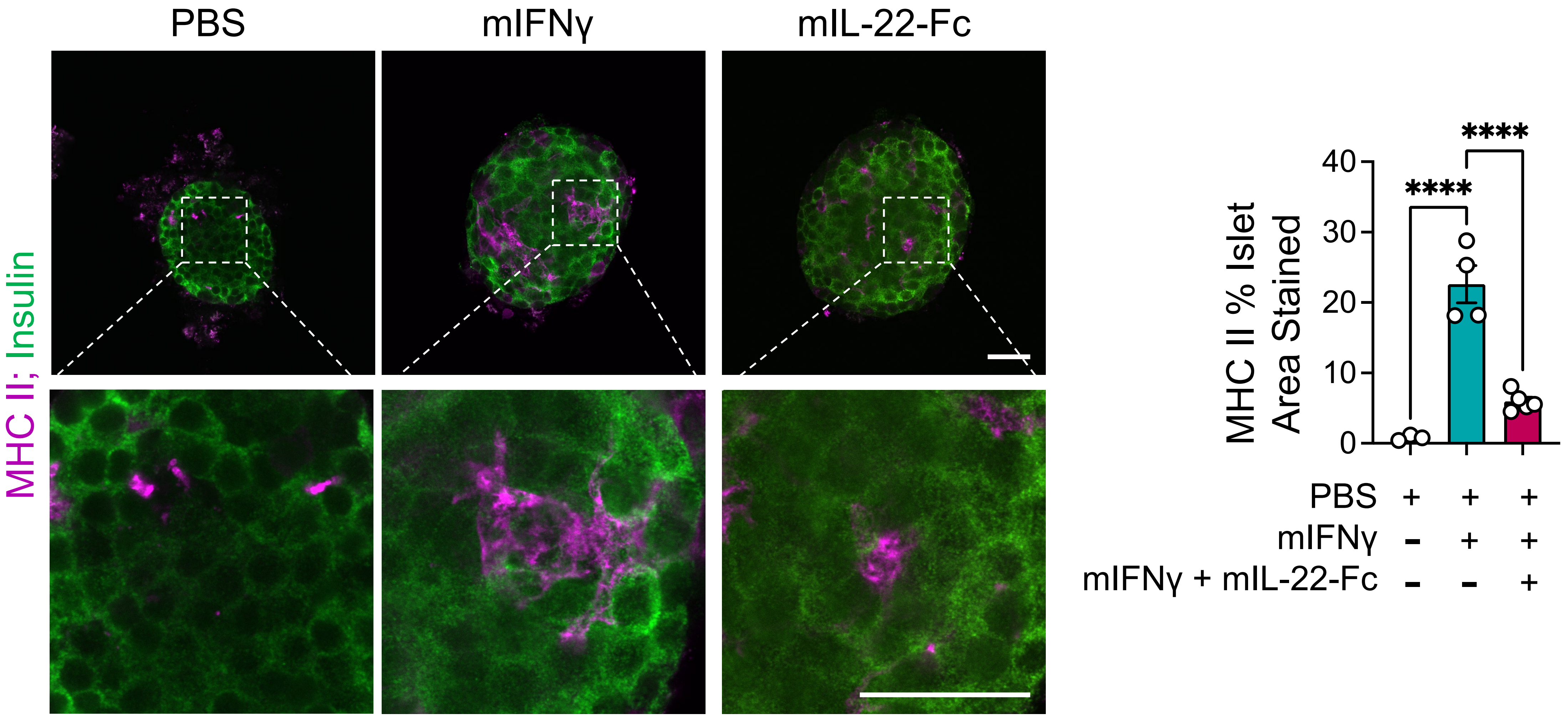

Supplementary Figure 10: IL-22 treatment reduced IFNγ induced pancreatic beta cell MHC II expression. MHC II and insulin co-staining in wildtype pancreatic islets following treatment with 10 ng mL^{-1} mIFNγ ± 50 ng mL^{-1} mIL-22-Fc for 48h. Graph is presented as Mean ± SEM. Female animals; n = 3 biologically independent islets from 4 pooled animals (PBS), n = 4 biologically independent islets from 4 pooled animals (mIFNγ), and n = 5 biologically independent islets from 4 pooled animals (mIFNγ + mIL-22-Fc). One way ANOVA. ****$p < 0.0001$; n.s., non-significant. Scale bar: 20 um. Source data are provided as a Source Data file.

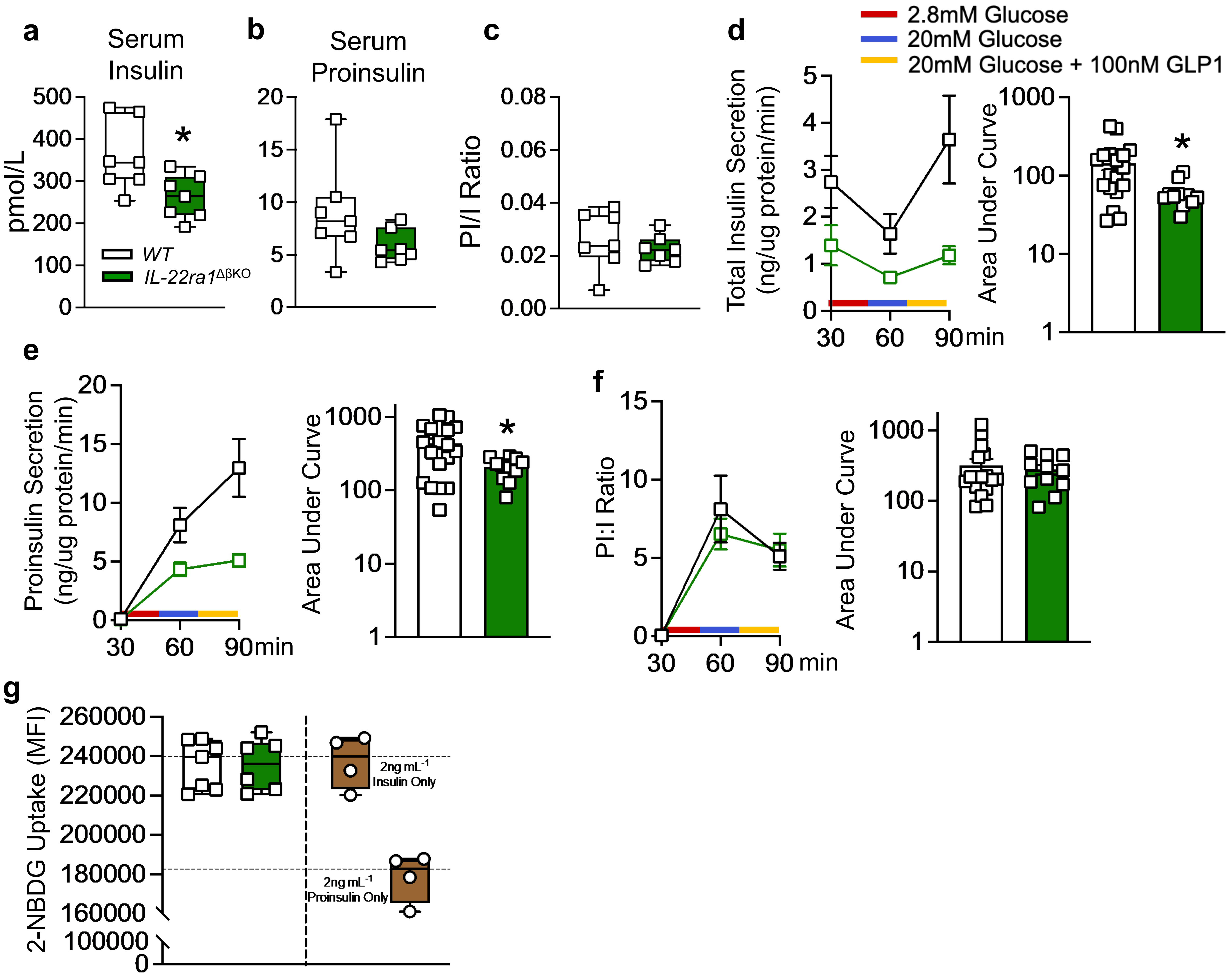

Supplementary Figure 11: In male animals, ablation of pancreatic β-cell IL-22ra1 signaling leads to reduced insulin biosynthesis (a) Serum total insulin (*p = 0.0265*), **(b)** proinsulin and **(c)** proinsulin : insulin ratio in animals at 20 weeks of age. **(d)** Total insulin secretion (ng/ug protein/min) *p = 0.0222*, **(e)** proinsulin secretion (ng/ug protein/min) *p = 0.0231*, and **(f)** proinsulin : insulin ratio of mouse islets during *in-vitro* glucose stimulated insulin secretion, following stimulation with 2.8 mM glucose, 20 mM glucose and 20mM glucose + 100nM GLP-1. **(g)** 2-NBDG uptake in 3T3-L1 adipocytes exposed to 2ng/mL islet insulin secretion following stimulation with 20mM glucose + 100nM GLP-1. Box plots in **(a-c, g)** display the median (central line), 25th to 75th percentile (box) and minimum to maximum values (whiskers). All other graphs are presented as Mean ± SEM. Male animals; **(a-c)** n = 7 biologically independent animals, **(d-g)** n = 18 independent samples (10 islets/sample) from 3 biologically independent wildtype (*IL-22ra$^{fl/fl}$*), and 11 independent samples (10 islets/sample) from 3 biologically independent *IL-22ra1$^{\Delta\beta KO}$* animals. Two-tailed unpaired Student's t-test. *p < 0.05; n.s., non-significant. *versus wildtype (*IL-22ra1$^{fl/fl}$*) littermate controls. Source data are provided as a Source Data file.

Table S1: Human Primers		
**Gene Name**	**Source**	**Catalogue Number**
IL-22RA1	Thermo Fisher Scientific	Hs00222035_m1; Cat # 4331182
IL-10RB	Thermo Fisher Scientific	Hs00175123_m1; Cat # 4331182
IL-20RA	Thermo Fisher Scientific	Hs01011609_m1; Cat # 4331182
IL-20RB	Thermo Fisher Scientific	Hs00376373_m1; Cat # 4331182
GAPDH	Thermo Fisher Scientific	Cat #4333764

Table S2: Mouse Primers		
**Gene Name**	**Forward Primer**	**Reverse Primer**
Cd68	TGTCTGATCTTGCTAGGACCG	GAGAGTAACGGCCTTTTTGTGA
Chop	GGAGGTCCTGTCCTCAGATGAA	GTCCTCTGTCAGCCAAGCTAG
Grp78	CGCCTCATCGGACGCACTTGG	GCTTGCCGCTGTGCATCATTGA
Socs3	GGACCAAGAACCTACGGCATCCA	CACCAGCTTGAGTACACAGTCC
Il1b	AGAGTTACACTGCCTTTGCCATCC	CCACGTCAATCTTTCCTCTTGCTT
Foxo3	CTGGGGGAACCTGTCCTATG	TCATTCTGAACGCGCATGAAG
Nos2	CAGCTGGGCTGTACAAACCTT	CATTGGAAGTGAAGCGTTTCG
sXbp1	GAGTCCGCAGCAGGTGC	CAAAAGGATATCAGACTCAGAATCTGAA
Pdx1	CCCCAGTTTACAAGCTCGCT	CTCGGTTCCATTCGGGAAAGG
Tnfa	CATCTTCTCAAAATTCGAGTGACAA	TGGGAGTAGACAAGGTACAACCC
!l6	TCAGGAAATTTGCCTATTGAAA	GGAAATTGGGGTAGGAAGGA
Ccl2	TTAAAAACCTGGATCGGAACCAA	GCATTAGCTTCAGATTTACGGGT
Sod2	AGACACGGCTGTCAG	CTGGACAAACCTGAG
Irs1	CGATGGCTTCTCAGACGTG	CAGCCCGCTTGTTGATGTTG
Irs2	CTGCGTCCTCTCCCAAAGTG	GGGGTCATGGGCATGTAGC
Mt1	AAGAGTGAGTTGGGACACCTT	CGAGACAATACAATGGCCTCC
Prkacb	CTCGGGACGGGTTCCTTTG	AGGGACGTATTCCATAACCATGT
Ywhaz	GAAAAGTTCTTGATCCCCAATGC	TGTGACTGGTCCACAATTCCTT
Neurod1	ATGACCAAATCATACAGCGAGAG	TCTGCCTCGTGTTCCTCGT
Neurog3	CCAAGAGCGAGTTGGCACT	CGGGCCATAGAAGCTGTGG
Reg3b	ACTCCCTGAAGAATATACCCTCC	CGCTATTGAGCACAGATACGAG
Reg3g	ATGCTTCCCCGTATAACCATCA	GGCCATATCTGCATCATACCAG
Pik3r1	ACACCACGGTTTGGACTATGG	GGCTACAGTAGTGGGCTTGG
Pik3r2	GGATGCCTGGCTTCAACGA	CTGGGAGTATGTGGCCTGACT
Eif4ebp1	GGGGACTACAGCACCACTC	CTCATCGCTGGTAGGGCTA
Rapgef4	CAAGGAGAATGTCCCTTCAGAGA	CCGCGAGTGAACACAGGAT
Mafa	AGGAGGAGGTCATCCGACTG	CTTCTCGCTCTCCAGAATGTG
Foxo1	CCCAGGCCGGAGTTTAACC	GTTGCTCATAAAGTCGGTGCT

Table S3: Human Islet Donor Demographics			
**Donor**	**Sex**	**Age**	**Medical History**
Healthy 1	F	61 - 65	No substantive history
Healthy 2	F	61 - 65	No substantive history
Healthy 3	M	66 - 70	No substantive history
T2D 1 (Fig. 1)	M	51 - 55	T2D, smoker, hypertensive
T2D 2 (Fig. 1)	F	66 - 70	T2D
T2D 3 (Fig. 1)	M	66 - 70	T2D, hypertensive
NGT 1	M	76 - 80	
NGT 2	F	31 - 35	
NGT 3	M	71 - 75	
NGT 4	F	76 - 80	
IGT 1	F	56 - 60	
IGT 2	M	46 - 50	
IGT 3	F	76 - 80	
IGT 4	F	76 - 80	
T2D 1 (Sup Fig.1)	F	71 - 75	T2D
T2D 2 (Sup Fig.1)	F	66 - 70	T2D
T2D 3 (Sup Fig.1)	F	71 - 75	T2D
T2D 4 (Sup Fig.1)	M	71 - 75	T2D
