# Supplementary material for: Pancreatic beta-cell IL-22 receptor deficiency induces age-dependent dysregulation of insulin biosynthesis and systemic glucose homeostasis
Source: Nat Commun. 2024 May 29;15:4527. doi: 10.1038/s41467-024-48320-2 (PMC11137127; doi:10.1038/s41467-024-48320-2)
Supplement: Supplementary file 3 — Reporting Summary [file 41467_2024_48320_MOESM3_ESM.pdf]

nature portfolio

Corresponding author(s): A/Prof Sumaira Zia Hasnain

Last updated by author(s): Apr 18, 2024

Reporting Summary

Nature Portfolio wishes to improve the reproducibility of the work that we publish. This form provides structure for consistency and transparency in reporting. For further information on Nature Portfolio policies, see our Editorial Policies and the Editorial Policy Checklist.

Statistics

For all statistical analyses, confirm that the following items are present in the figure legend, table legend, main text, or Methods section.

n/a	Confirmed	
☐	☒	The exact sample size (n) for each experimental group/condition, given as a discrete number and unit of measurement
☐	☒	A statement on whether measurements were taken from distinct samples or whether the same sample was measured repeatedly
☐	☒	The statistical test(s) used AND whether they are one- or two-sided<br>*Only common tests should be described solely by name; describe more complex techniques in the Methods section.*
☐	☒	A description of all covariates tested
☐	☒	A description of any assumptions or corrections, such as tests of normality and adjustment for multiple comparisons
☐	☒	A full description of the statistical parameters including central tendency (e.g. means) or other basic estimates (e.g. regression coefficient) AND variation (e.g. standard deviation) or associated estimates of uncertainty (e.g. confidence intervals)
☐	☒	For null hypothesis testing, the test statistic (e.g. F, t, r) with confidence intervals, effect sizes, degrees of freedom and P value noted<br>*Give P values as exact values whenever suitable.*
☒	☐	For Bayesian analysis, information on the choice of priors and Markov chain Monte Carlo settings
☒	☐	For hierarchical and complex designs, identification of the appropriate level for tests and full reporting of outcomes
☒	☐	Estimates of effect sizes (e.g. Cohen's d, Pearson's r), indicating how they were calculated

Our web collection on statistics for biologists contains articles on many of the points above.

Software and code

Policy information about availability of computer code

Data collection	Immuno-flourescence microscopy was performed on the Olympus FV3000 Confocal Laser Scanning Microscope and data was collected using the FV31S-SW Viewer Software V2.6; qRT-PCR data was collected using the QuantStudio Real-Time PCR Software V1.7.2; PHERAstar Plus V5.70 (Plate Reader)
Data analysis	Qiagen Ingenuity Pathway Analysis Software 2023 winter version, MARS Data Analysis Software V4.01 R2 (Plate Reader), Graphpad Prism V10.2.2, ImageJ V1.54i, Visiopharm V2018.4

For manuscripts utilizing custom algorithms or software that are central to the research but not yet described in published literature, software must be made available to editors and reviewers. We strongly encourage code deposition in a community repository (e.g. GitHub). See the Nature Portfolio guidelines for submitting code & software for further information.

Data

Policy information about availability of data

All manuscripts must include a data availability statement. This statement should provide the following information, where applicable:

- Accession codes, unique identifiers, or web links for publicly available datasets
- A description of any restrictions on data availability
- For clinical datasets or third party data, please ensure that the statement adheres to our policy

The RNA-Seq dataset generated and analysed during the current study is available on the NCBI Gene Expression Omnibus (GEO) database, under accession code GSE262867. All other data generated in this study are provided in the Supplementary Information/Source Data file. Source data are provided with this paper.

Research involving human participants, their data, or biological material

Policy information about studies with human participants or human data. See also policy information about sex, gender (identity/presentation), and sexual orientation and race, ethnicity and racism.

Reporting on sex and gender	For the experiment in Figure 1, islets were isolated from 3 male and 3 female organ donors. For experiment in Figure Supplementary Figure 1, pancreatic islets were isolated from 4 male and 8 female patients undergoing pylorus-preserving pancreatoduodenectomy.
Reporting on race, ethnicity, or other socially relevant groupings	N/A
Population characteristics	The demographic profile of participants in Figure 1 includes six individuals, divided into two categories: Healthy and those with Type 2 Diabetes (T2D). The Healthy group is composed of two females and one male, each aged between 61 and 65 years, with no substantive medical history reported. The T2D group comprises two males and one female, spanning an age range from 51 to 70 years. Within this group, one male aged 51-55 years is noted to be a smoker and hypertensive, another male aged 66-70 years is hypertensive, and the female aged 66-70 years does not have additional conditions besides T2D.<br><br>The dataset in Supplementary Figure 1 encompasses twelve individuals, categorized into three groups based on glucose tolerance status: Normal Glucose Tolerance (NGT), Impaired Glucose Tolerance (IGT), and Type 2 Diabetes (T2D). The NGT group consists of four individuals: two males and two females, covering a significant age spread from 31 to 80 years. Specific ages include one female in the youngest bracket of 31-35 years and the rest in the senior range of 71-80 years. The IGT group also comprises four individuals, with three females and one male participant. The ages in this group range from 46 to 80 years, with the females prominently in the upper age bracket of 56-80 years. Participants in the NGT and IGT groups have no relevant significant medical history. Lastly, the T2D group includes three females and one male, all between 66 and 75 years of age. All members of this group have been diagnosed with Type 2 Diabetes.
Recruitment	Islet samples in Figure 1 were obtained from deceased organ donors via the Tom Mandel Islet Transplant Program in Australia. For the experiment in Supplementary Figure 1, patients undergoing pylorus-preserving pancreatoduodenectomy were recruited from January 2017 to July 2019 at the Digestive Surgery Unit and studied at the Centre for Endocrine and Metabolic Diseases unit (Agostino Gemelli University Hospital, Rome, Italy). All patients underwent complete metabolic phenotyping including OGTT and mixed meal test (MMT). Participants were metabolically profiled prior to surgery;based on thresholds set by the ADA for fasting glucose, HbA1c and 2 h glucose level during an OGTT in the days immediately before surgery, participants were then classified as NGT (n=4), IGT (n=4) or with disease onset longer than 1 year; T2D (n=4).
Ethics oversight	The human islet procurement and experimental protocols in this study received approval from the Mater Health Services Human Research Ethics Committee (ER Stress in Pancreatic Islet – HREC/MML/23899). which provided the ethical oversight.<br><br>The study protocol for human islet experiments in Supplementary Fig. 1 (ClinicalTrials.gov registration no. NCT02175459), was approved by the local ethics committee (P/656/CE2010 and 22573/14) (Rome, Italy) and all participants provided written informed consent, which was followed by a comprehensive medical evaluation.

Note that full information on the approval of the study protocol must also be provided in the manuscript.

Field-specific reporting

Please select the one below that is the best fit for your research. If you are not sure, read the appropriate sections before making your selection.

☒ Life sciences ☐ Behavioural & social sciences ☐ Ecological, evolutionary & environmental sciences

For a reference copy of the document with all sections, see nature.com/documents/nr-reporting-summary-flat.pdf

Life sciences study design

All studies must disclose on these points even when the disclosure is negative.

Sample size	Power calculation conducted by our biostatistician, based on previous data, showed that studies required a sample size of n = 3-12 to achieve a power of 0.8 with an alpha value of 0.05 to detect a true difference in means between the treatment group and control

	group of 20%.
Data exclusions	Data was excluded based on technical error eg. high background on staining, unacceptable qRT-PCR melt curves
Replication	All experiments were repeated, all attempts at replication were successful
Randomization	All cells/animals in this experiment were randomly allocated to a treatment group.
Blinding	Investigators were blinded to group allocation during data analysis.

Reporting for specific materials, systems and methods

We require information from authors about some types of materials, experimental systems and methods used in many studies. Here, indicate whether each material, system or method listed is relevant to your study. If you are not sure if a list item applies to your research, read the appropriate section before selecting a response.

Materials & experimental systems

n/a	Involved in the study
☐	☒ Antibodies
☐	☒ Eukaryotic cell lines
☒	☐ Palaeontology and archaeology
☐	☒ Animals and other organisms
☒	☐ Clinical data
☒	☐ Dual use research of concern
☒	☐ Plants

Methods

n/a	Involved in the study
☒	☐ ChIP-seq
☒	☐ Flow cytometry
☒	☐ MRI-based neuroimaging

Antibodies

Antibodies used

Rabbit anti-Iba1 (Novachem, Cat # S03866; 1:1000)
Rat anti-IL-22ra1 (R&D, Cat # MAB-42941; 1:100)
Mouse anti-proinsulin (R&D, Cat # MAB-13361; 1:200)
Guinea Pig anti-insulin (Invitrogen, Cat # PA-26938; 1:500)
Rabbit anti-Grp78 (Sigma Aldrich, Cat # 8918; 1:1000)
Mouse anti-4-Hne (Invitrogen, Cat # MA5-27570, 1:200)
Rat anti-MHC II (Invitrogen, Cat # 14-5321-82; 1:500)
Rabbit anti-Ki-67 (Invitrogen, Cat # MA5-14520; 1:500)
Rabbit anti-Cre recombinase (Cell Signaling Technology, Cat # 15036; 1:200)
AF488 Goat anti-Guinea Pig (Invitrogen, Cat # A-11073; 1:500)
AF555 Goat anti-Rabbit (Invitrogen, Cat # A-32732; 1:500)
AF647 Goat anti Rabbit (Invitrogen, Cat # A-32733, 1:500)
AF555 Goat anti Rat (Invitrogen, Cat # A-21434; 1:500)
AF555 Goat anti Mouse (Invitrogen, Cat # A-21422; 1:500)

Validation

All antibodies used in this study are commercially available and have been validated by the manufacturer and/or other investigators as indicated on the websites:
Rabbit anti-Iba1: https://www.novachem.com.au/shop/019-19741-anti-iba1-rabbit-for-immunocytochemistry-306642#attr=
Rat anti-IL-22ra1: https://www.rndsystems.com/products/mouse-il-22-ralpha1-antibody-496514_mab42941
Mouse anti-proinsulin: https://www.rndsystems.com/products/human-mouse-proinsulin-antibody-253627_mab13361
Guinea Pig anti-insulin: https://www.thermofisher.com/antibody/product/Insulin-Antibody-Polyclonal/PA1-26938
Rabbit anti-Grp78: https://www.sigmaaldrich.com/AU/en/product/sigma/g8918
Mouse anti-4-Hne: https://www.thermofisher.com/antibody/product/4-Hydroxynonenal-Antibody-clone-12F7-Monoclonal/MA5-27570
Rat anti-MHC II: https://www.thermofisher.com/antibody/product/MHC-Class-II-I-A-I-E-Antibody-clone-M5-114-15-2-Monoclonal/14-5321-82
Rabbit anti-Ki-67: https://www.thermofisher.com/antibody/product/Ki-67-Antibody-clone-SP6-Recombinant-Monoclonal/MA5-14520
Rabbit anti-Cre recombinase: https://www.cellsignal.com/products/primary-antibodies/cre-recombinase-d7l7l-xp-rabbit-mab/15036
AF488 Goat anti-Guinea Pig: https://www.thermofisher.com/antibody/product/Goat-anti-Guinea-Pig-IgG-H-L-Highly-Cross-Adsorbed-Secondary-Antibody-Polyclonal/A-11073
AF555 Goat anti-Rabbit: https://www.thermofisher.com/antibody/product/Goat-anti-Rabbit-IgG-H-L-Highly-Cross-Adsorbed-Secondary-Antibody-Polyclonal/A32732
AF647 Goat anti Rabbit: https://www.thermofisher.com/antibody/product/Goat-anti-Rabbit-IgG-H-L-Highly-Cross-Adsorbed-Secondary-Antibody-Polyclonal/A32733
AF555 Goat anti Rat: https://www.thermofisher.com/antibody/product/Goat-anti-Rat-IgG-H-L-Cross-Adsorbed-Secondary-Antibody-Polyclonal/A-21434
AF555 Goat anti Mouse: https://www.thermofisher.com/antibody/product/Goat-anti-Mouse-IgG-H-L-Cross-Adsorbed-Secondary-

Antibody-Polyclonal/A-21422

Eukaryotic cell lines

Policy information about cell lines and Sex and Gender in Research

Cell line source(s)	3T3-L1 Cells - ATCC, Manassas, Virginia, USA; CL-173<br>MIN6N8 cells - kind gift from J. Miyazaki, Osaka University
Authentication	Cell lines were authenticated by morphology check under the microscope prior to each experiment.
Mycoplasma contamination	All cell lines tested negative for mycoplasma contamination
Commonly misidentified lines<br>(See ICLAC register)	No commonly misidentified cell lines were used.

Animals and other research organisms

Policy information about studies involving animals; ARRIVE guidelines recommended for reporting animal research, and Sex and Gender in Research

Laboratory animals	IL-22ra1 fl/fl x Ins2-cre male and female animals, 8 weeks old at start of experiments.<br>IL-22ra1 fl/fl x Gcg-cre male and female animals, 8 weeks old at start of experiments
Wild animals	This study did not involve wild animals
Reporting on sex	Both male and female animals were utilized in this study
Field-collected samples	This study did not involve samples collected from the field
Ethics oversight	All animal experiments were approved by the University of Queensland Animal Ethics Committee (Ethics #2021/AE000426, AE519/16) and conducted in accordance with guidelines set out by the National Health and Medical Research Council of Australia.

Note that full information on the approval of the study protocol must also be provided in the manuscript.

Plants

Seed stocks	N/A
Novel plant genotypes	N/A
Authentication	N/A
